# Impact of China's National Volume‐Based Procurement Policy on Cost, Volume, and Market Structure: An Interrupted Time‐Series Analysis of Montelukast Sodium

**DOI:** 10.1002/hcs2.70092

**Published:** 2026-08-06

**Authors:** Yufei Cheng, Fanyu Liu, Yongyi Wu, Siyu Liu, Xiaohan Fan, Bin Jiang, Zhao Yang

**Affiliations:** ^1^ School of Population and Health Renmin University of China Beijing China; ^2^ Institute of New Structural Economics Peking University Beijing China; ^3^ School of Public Health Kunming Medical University Kunming China; ^4^ School of Public Health China Medical University Shenyang China; ^5^ Scientific Research Department Peking University First Hospital Beijing China; ^6^ Research Center of Public Policy Peking University Beijing China; ^7^ School of Pharmaceutical Sciences Peking University Beijing China; ^8^ Center for Digital Health and Artificial Intelligence Peking University First Hospital Beijing China

**Keywords:** drug policy, interrupted time‐series analysis, montelukast sodium, National Volume‐Based Procurement, price control

## Abstract

**Background:**

To reduce drug prices and improve access to essential medicines, the Chinese government implemented the National Volume‐Based Procurement (NVBP) policy, leveraging economies of scale. Montelukast sodium, widely prescribed for respiratory diseases, was included in the first round. This study evaluates the impact of the NVBP policy, offering insights for global pharmaceutical policy reform.

**Methods:**

Utilizing national procurement data from the National Healthcare Security Administration (January 2019 to December 2020), we conducted an interrupted time‐series analysis, employing autoregressive integrated moving average models to correct for autocorrelation. We evaluated changes in procurement volume (in defined daily doses), procurement expenditures, and defined daily dose costs of montelukast sodium before and after NVBP implementation.

**Results:**

The NVBP policy triggered a substantial decline in total procurement expenditures and defined daily dose costs, significantly improving drug affordability. Unlike the volume surge observed in other NVBP‐covered drugs, the total procurement volume of montelukast sodium remained stable, suggesting rational clinical demand. Structurally, the policy successfully drove a “generic substitution” effect, where bid‐winning generics rapidly replaced high‐priced originators. The market share of non‐equivalent generics contracted significantly, marking a quality consolidation of the market.

**Conclusions:**

The NVBP successfully achieved cost containment for chronic respiratory disease medications. By promoting high‐quality generic substitution and reducing the market share of high‐priced originators and low‐quality generics, the policy effectively optimized the market structure. These findings provide a replicable China Model for low‐ and middle‐income countries seeking to balance affordability and accessibility in chronic disease management.

## Graphical abstract

Using ARIMA‐based interrupted time‐series analysis, this study reveals that China's NVBP policy significantly reduced daily costs and enhanced affordability, driving generic substitution and the elimination of low‐quality products. Additionally, non‐winning manufacturers adopted strategic pricing behaviors to segment the market.

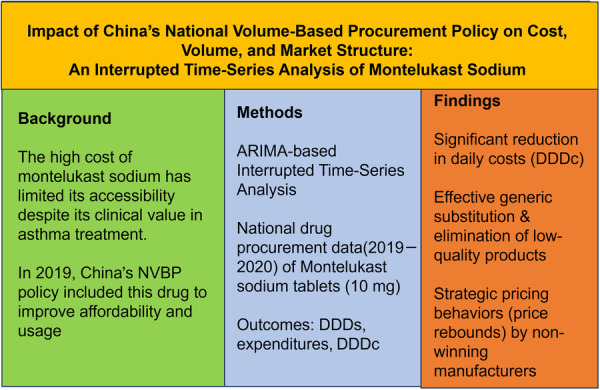

**Abbreviations**
ARIMA: autoregressive integrated moving average
CNY: Chinese Yuan
DDD: defined daily dose
DDDc: defined daily dose cost
ITS: interrupted time‐series
MSD: Merck Sharp & Dohme
NVBP: National Volume‐Based Procurement

## 1 Background

Asthma is a pervasive chronic respiratory disease, with prevalence ranging from 1% to 18% globally [1]. Affecting approximately 262 million people in 2019, it places a substantial health and economic burden on society [2, 3]. Unlike acute conditions, asthma requires long‐term, continuous management to prevent exacerbations. Consequently, the financial burden of medication is a critical determinant of treatment adherence and control [4]. In China, asthma prevalence has risen steadily, with medication costs accounting for the largest proportion (61.45%) of total treatment expenditures [5]. As a key maintenance therapy, montelukast sodium—a potent leukotriene receptor antagonist—is widely prescribed due to its efficacy and lack of tolerance issues [6]. However, it has historically remained expensive. Despite the patent expiration of the originator brand (Singulair, Merck Sharp & Dohme [MSD]) in 2012, the lack of high‐quality generic competition kept prices high (6–7 Chinese Yuan [CNY] per tablet) for years in China, mirroring the high costs and rising price trends observed in the United States and Europe [7, 8, 9].

To dismantle such price barriers and improve accessibility, China introduced the National Volume‐Based Procurement (NVBP) policy in 2019. Similar to group purchasing organizations in the United States and tendering systems in the European Union [10, 11, 12, 13], the NVBP leverages the consolidation of national demand to trade “volume for price.” While numerous studies have evaluated the NVBP's impact on cardiovascular drugs [14, 15], antineoplastic agents [16, 17], and antibiotics [18], evidence regarding its impact on chronic respiratory maintenance medications remains limited. Furthermore, existing literature largely focuses on whether overall costs have decreased, often overlooking the micro‐level structural reshaping of the market—specifically, how the policy affects the complex interplay between bid‐winning products, non‐winning products, originator drugs, qualified generics, and non‐equivalent products, as well as the strategic pricing behaviors of various manufacturers.

In December 2019, the NVBP included montelukast sodium (10 mg) in its procurement list, marking a pivotal shift in the market structure. Prior to this, MSD dominated the market. Upon policy implementation, only MSD (the originator) and Anbison (the first generic to pass the Generic Consistency Evaluation) won the bid, with prices drastically cut to 3.88 and 3.79 CNY per tablet, respectively [19]. Other manufacturers, such as Organon, Sichuan Otsuka, and Lunan Better, either failed to win the bid or were unqualified at the time due to pending consistency evaluations. However, these products remained in circulation, creating a distinct “dual‐track” market where bid‐winning and non‐winning products co‐existed. This scenario provides an ideal case to analyze the structural effects of the policy.

Therefore, utilizing interrupted time‐series (ITS) analysis based on data from the pre‐policy period (January–December 2019) and the post‐policy period (January–December 2020), this study aims to evaluate the impact of the NVBP on the procurement volume, expenditures, and defined daily dose cost (DDDc) of montelukast sodium. Beyond these aggregate metrics, we specifically investigate the following: (1) structural reshaping of the market, including the generic substitution effect and the market exit of low‐quality products, and (2) the strategic pricing behaviors of non‐winning products. This study not only fills the literature gap regarding the NVBP in the field of respiratory chronic diseases but also provides empirical evidence for future policy optimization.

## 2 Methods

### 2.1 Data

This study conducted a retrospective analysis of pharmaceutical procurement data to evaluate the impact of the NVBP policy on montelukast sodium. Data were obtained from the National Healthcare Security Administration database, which aggregates procurement records from public healthcare providers across China. The study period spanned 24 months, from January 1, 2019, to December 31, 2020. The dataset encompassed procurement records from approximately 1.115 million healthcare facilities across 25 provincial‐level administrative regions. This extensive sample included tertiary hospitals, secondary hospitals, and primary healthcare institutions (e.g., community health centers), covering a population of over 762 million. This broad coverage ensures high national representativeness and allows for a comprehensive assessment of policy effects across different tiers of the healthcare delivery system. Each procurement record contained detailed information, including the transaction date, province, generic name, dosage form, manufacturer, procurement volume, and expenditures.

### 2.2 Outcomes

Three outcome measures were assessed: procurement volume, procurement expenditures, and DDDc. Procurement volume was measured in thousand defined daily doses (DDDs). The total DDDs were calculated as the total quantity of the drug (in mg) divided by its specific DDD, as standardized by the World Health Organization [20]. Procurement expenditures were reported in million CNY. DDDc was used to measure the price level, calculated as the total procurement expenditures divided by the total DDDs (expenditure per DDD). This metric reflects the average daily treatment cost and accounts for changes in both price and packaging specifications.

### 2.3 Statistical Analysis

We employed an ITS design to analyze the impact of the NVBP policy on the usage of montelukast sodium. ITS is a quasi‐experimental design that evaluates the longitudinal effects of an intervention by comparing trends before and after the event. The segmented regression model is specified as Formula (1).

(1) $$Y_{t}=\beta_{0}+\beta_{1}\times {\text{Time}}_{t}+\beta_{2}\times {\text{Policy}}_{t}+\beta_{3}\times {\text{Trend}}_{t}+\varepsilon_{t}$$

where

$Y_{t}$ represents the outcome variables (DDDs, expenditures, or DDDc) in month t;

$\beta_{0}$ is the intercept (baseline level);

$\beta_{1}$ estimates the baseline slope prior to the intervention;

${\mathrm{Time}}_{t}$ is a continuous variable indicating the time in months from the start of the study period;

$\beta_{2}$ estimates the immediate change in the level of the outcome following the intervention;

${\mathrm{Policy}}_{t}$ is a binary indicator variable, coded as 0 before the intervention and 1 after the implementation of NVBP (from December 2019 onwards);

$\beta_{3}$ estimates the change in the trend (slope) after the intervention;

${\mathrm{Trend}}_{t}$ is a continuous variable counting the number of months since the intervention (coded as 0 before December 2019); and

$\varepsilon_{t}$ is the error term.

Since time‐series data often exhibit autocorrelation, which violates the independence assumption of ordinary least squares regression, we employed autoregressive integrated moving average (ARIMA) models for the analysis. We tested multiple ARIMA specifications, including ARIMA (1, 0, 0), ARIMA (0, 0, 1), and ARIMA (1, 0, 1), and evaluated model fit based on the Akaike information criterion and Bayesian information criterion. The Ljung–Box *Q* test was performed post‐estimation to confirm that the residuals behaved as white noise. Ultimately, the ARIMA (1, 0, 0) model was adopted for the final analysis, as it successfully corrected for autocorrelation (Ljung–Box *p* > 0.05) across the datasets, ensuring methodological consistency.

## 3 Results

### 3.1 Procurement Volume

**Figure 1:**
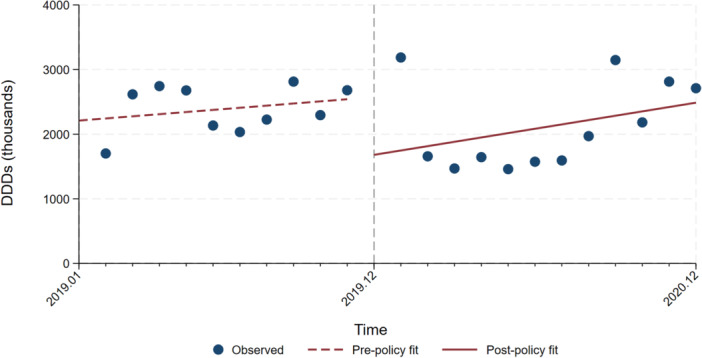
Monthly trends in the procurement volume of montelukast sodium in China (2019–2020). This figure presents a nationwide ITS analysis of montelukast sodium procurement. The dots represent the observed monthly procurement volume. The dashed and solid lines represent the pre‐policy and post‐policy trends, respectively, estimated by the ITS model. The vertical dashed line indicates the implementation of the NVBP policy (December 2019). DDD, defined daily dose; ITS, interrupted time‐series.

**Figure 2:**
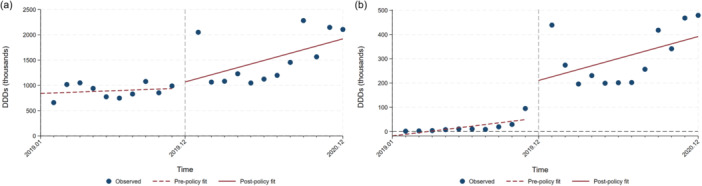
Monthly trends in the procurement volume of originator and generic montelukast sodium in China (2019–2020). This figure presents ITS analyses of montelukast sodium procurement. Panel (a) displays the trends for originator drugs, and panel (b) displays the trends for generic drugs. The dots represent the observed monthly procurement volume. The dashed and solid lines represent the pre‐policy and post‐policy trends, respectively, estimated by the ITS model. The vertical dashed line indicates the implementation of the NVBP policy (December 2019). DDD, defined daily dose; ITS, interrupted time‐series; NVBP, National Volume‐Based Procurement.

**Figure 3:**
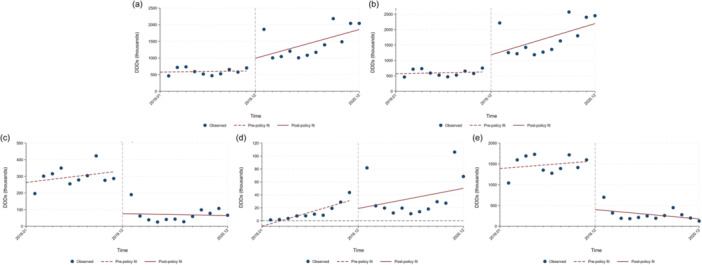
Monthly trends in the procurement volume of montelukast sodium across different subgroups in China (2019–2020). This figure presents ITS analyses of montelukast sodium procurement across different subgroups. Panel (a) displays the trends for bid‐winning originator drugs, panel (b) for bid‐winning generic drugs, panel (c) for non‐winning originator drugs, panel (d) for non‐winning generic drugs, and panel (e) for non‐equivalent generic drugs. The dots represent the observed monthly procurement volume. The dashed and solid lines represent the pre‐policy and post‐policy trends, respectively, estimated by the ITS model. The vertical dashed line indicates the implementation of the NVBP policy (December 2019). DDD, defined daily dose; ITS, interrupted time‐series; NVBP, National Volume‐Based Procurement.

The monthly trends in the procurement volume of montelukast sodium are displayed in Figures 1, 2, 3. Table 1 presents the results of the ITS analysis, with estimates adjusted for autocorrelation using ARIMA models.

**Table 1:** Impact of the NVBP policy on the monthly procurement volume of montelukast sodium in China (2019–2020).

Group	Baseline level (*β*_0_)	Baseline trend (*β*_1_)	Level change (*β*_2_)	Trend change (*β*_3_)
Nationwide	2116.540^b^	39.594	−807.379	14.655
Originator drugs	822.617^a^	10.653	85.576	57.562
Generic drugs	−11.449	2.815	249.602^a^	6.760
Bid‐winning originator drugs	570.419	3.744	320.869	66.989
Bid‐winning generic drugs	550.576	7.220	532.437	71.290
Non‐winning originator drugs	256.305^b^	6.478	−249.333^b^	−7.575
Non‐winning generic drugs	−9.953	3.488	−1.248	−1.836
Non‐equivalent generic drugs	1331.992^b^	21.426	−1117.341^b^	−43.589

*Note:* Estimates were derived from ITS models adjusted for autocorrelation using ARIMA specifications. Data are presented in thousand DDDs.

Abbreviations: ARIMA, autoregressive integrated moving average; DDD, defined daily dose; ITS, interrupted time‐series; NVBP, National Volume‐Based Procurement.

^a^ *p* < 0.10,

^b^ *p* < 0.01.

At the national level, as shown in Figure 1, the implementation of the NVBP policy did not result in a statistically significant immediate change in the procurement volume of montelukast sodium or a significant shift in the long‐term trend (*p* > 0.1). This suggests that the overall procurement volume of the drug remained relatively stable following the policy intervention.

Regarding the comparison between originator and generic drugs (Figure 2), generic drugs experienced an immediate increase in procurement volume following the policy implementation, rising by 249.602 thousand DDDs, which was significant at the 10% level (*β*_2_ = 249.602, *p* < 0.1). In contrast, neither the immediate level change nor the long‐term trend change for originator drugs reached statistical significance (*p* > 0.1).

At the subgroup level (Figure 3), the analysis of bid‐winning and non‐winning drugs reveals significant structural differences. For bid‐winning drugs, neither bid‐winning originator nor bid‐winning generic drugs showed statistically significant immediate level changes or long‐term trend shifts in procurement volume following the policy implementation (*p* > 0.1). Conversely, regarding non‐winning drugs, non‐winning originator drugs experienced a significant immediate decline in procurement volume, decreasing by 249.333 thousand DDDs (*β*_2_ = −249.333, *p* < 0.01). For non‐winning generic drugs, no statistically significant changes were observed in either the immediate level or the long‐term trend (*p* > 0.1). Notably, non‐equivalent generic drugs experienced the most substantial significant decline, with an immediate reduction of 1117.341 thousand DDDs (*β*_2_ = −1117.341, *p* < 0.01). This reflects a sharp contraction in the market share of these drugs following the policy implementation.

### 3.2 Procurement Expenditure

**Figure 4:**
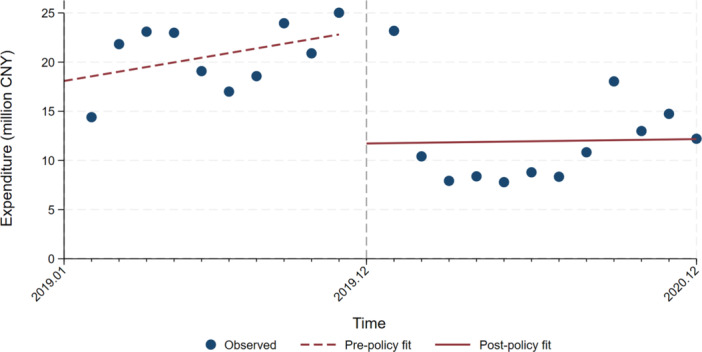
Monthly trends in the procurement expenditure of montelukast sodium in China (2019–2020). This figure presents a nationwide ITS analysis of montelukast sodium procurement. The dots represent the observed monthly procurement expenditure. The dashed and solid lines represent the pre‐policy and post‐policy trends, respectively, estimated by the ITS model. The vertical dashed line indicates the implementation of the NVBP policy (December 2019). CNY, Chinese Yuan; ITS, interrupted time‐series; NVBP, National Volume‐Based Procurement.

**Figure 5:**
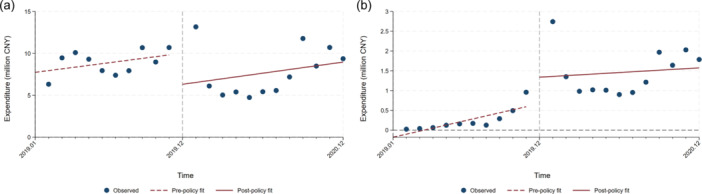
Monthly trends in the procurement expenditure of originator and generic montelukast sodium in China (2019–2020). This figure presents ITS analyses of montelukast sodium procurement. Panel (a) displays the trends for originator drugs, and panel (b) displays the trends for generic drugs. The dots represent the observed monthly procurement expenditure. The dashed and solid lines represent the pre‐policy and post‐policy trends, respectively, estimated by the ITS model. The vertical dashed line indicates the implementation of the NVBP policy (December 2019). CNY, Chinese Yuan; ITS, interrupted time‐series; NVBP, National Volume‐Based Procurement.

**Figure 6:**
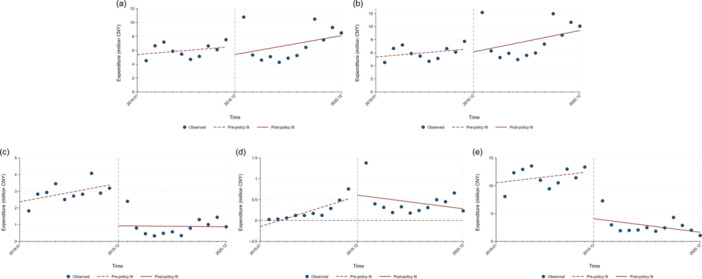
Monthly trends in the procurement expenditure of montelukast sodium across different subgroups in China (2019–2020). This figure presents ITS analyses of montelukast sodium procurement across different subgroups. Panel (a) displays the trends for bid‐winning originator drugs, panel (b) for bid‐winning generic drugs, panel (c) for non‐winning originator drugs, panel (d) for non‐winning generic drugs, and panel (e) for non‐equivalent generic drugs. The dots represent the observed monthly procurement expenditure. The dashed and solid lines represent the pre‐policy and post‐policy trends, respectively, estimated by the ITS model. The vertical dashed line indicates the implementation of the NVBP policy (December 2019). CNY, Chinese Yuan; ITS, interrupted time‐series; NVBP, National Volume‐Based Procurement.

The monthly trends in the procurement expenditure of montelukast sodium are displayed in Figures 4, 5, 6. Table 2 presents the results of the ITS analysis, with estimates adjusted for autocorrelation using ARIMA models.

**Table 2:** Impact of the NVBP policy on the monthly procurement expenditure of montelukast sodium in China (2019–2020).

Group	Baseline level (*β*_0_)	Baseline trend (*β*_1_)	Level change (*β*_2_)	Trend change (*β*_3_)
Nationwide	16.659^c^	0.561	−8.744^a^	−0.776
Originator drugs	7.264^b^	0.222	−2.330	−0.132
Generic drugs	−0.174	0.056	1.413^b^	−0.087
Bid‐winning originator drugs	5.069^a^	0.120	−0.239	0.005
Bid‐winning generic drugs	5.031	0.125	0.633	0.028
Non‐winning originator drugs	2.241^c^	0.103	−2.378^c^	−0.115
Non‐winning generic drugs	−0.172	0.059	0.398	−0.112
Non‐equivalent generic drugs	9.772^c^	0.256	−7.723^c^	−0.526^a^

*Note:* Estimates were derived from ITS models adjusted for autocorrelation using ARIMA specifications. Data are presented in million CNY.

Abbreviations: ARIMA, autoregressive integrated moving average; CNY, Chinese Yuan; ITS, interrupted time‐series; NVBP, National Volume‐Based Procurement.

^a^ *p* < 0.10,

^b^ *p* < 0.05,

^c^ *p* < 0.01.

At the national level, as shown in Figure 4, the implementation of the NVBP policy resulted in an immediate decline in procurement expenditure of 8.744 million CNY, which was significant at the 10% level (*β*_2_ = −8.744, *p* < 0.1); however, no significant change was observed in the long‐term trend (*p* > 0.1).

Regarding the comparison between originator and generic drugs (Figure 5), generic drugs experienced a statistically significant immediate increase in procurement expenditure following the policy implementation, rising by 1.413 million CNY (*β*_2_ = 1.413, *p* < 0.05). In contrast, neither the immediate level change nor the long‐term trend change for originator drugs reached statistical significance (*p* > 0.1).

At the subgroup level (Figure 6), the analysis of bid‐winning and non‐winning drugs reveals that changes in procurement expenditure were primarily driven by the contraction of non‐winning drugs. For bid‐winning drugs, neither bid‐winning originator nor bid‐winning generic drugs showed statistically significant immediate level changes or long‐term trend shifts in procurement expenditure (*p* > 0.1). Conversely, regarding non‐winning drugs, non‐winning originator drugs experienced a significant immediate decline in expenditure, decreasing by 2.378 million CNY (*β*_2_ = −2.378, *p* < 0.01). Notably, non‐equivalent generic drugs experienced the most substantial significant decline, with an immediate reduction of 7.723 million CNY (*β*_2_ = −7.723, *p* < 0.01). Furthermore, this category exhibited a downward trend in the long term that was significant at the 10% level, decreasing by 0.526 million CNY per month (*β*_3_ = −0.526, *p* < 0.1).

### 3.3 DDDc

**Figure 7:**
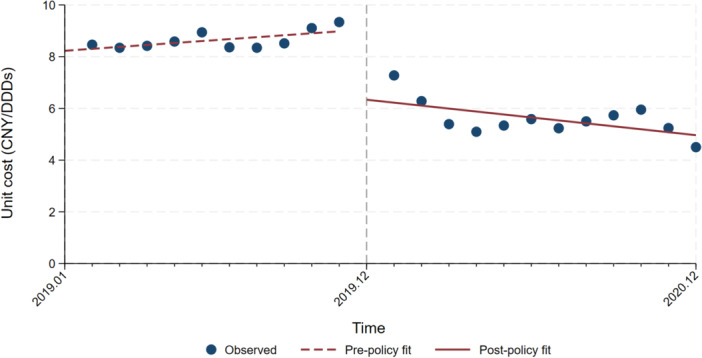
Monthly trends in the DDDc of montelukast sodium in China (2019–2020). This figure presents a nationwide ITS analysis of montelukast sodium procurement. The dots represent the observed monthly DDDc. The dashed and solid lines represent the pre‐policy and post‐policy trends, respectively, estimated by the ITS model. The vertical dashed line indicates the implementation of the NVBP policy (December 2019). CNY, Chinese Yuan; DDD, defined daily dose; DDDc, defined daily dose cost; ITS, interrupted time‐series; NVBP, National Volume‐Based Procurement.

**Figure 8:**
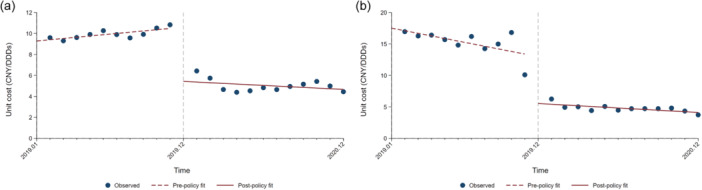
Monthly trends in the DDDc of originator and generic montelukast sodium in China (2019–2020). This figure presents ITS analyses of montelukast sodium procurement. Panel (a) displays the trends for originator drugs, and panel (b) displays the trends for generic drugs. The dots represent the observed monthly DDDc. The dashed and solid lines represent the pre‐policy and post‐policy trends, respectively, estimated by the ITS model. The vertical dashed line indicates the implementation of the NVBP policy (December 2019). CNY, Chinese Yuan; DDD, defined daily dose; DDDc, defined daily dose cost; ITS, interrupted time‐series; NVBP, National Volume‐Based Procurement.

**Figure 9:**
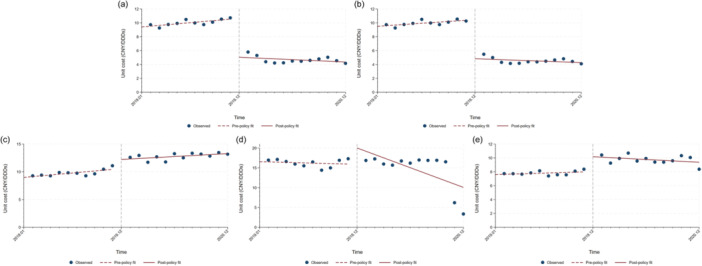
Monthly trends in the DDDc of montelukast sodium across different subgroups in China (2019–2020). This figure presents ITS analyses of montelukast sodium procurement across different subgroups. Panel (a) displays the trends for bid‐winning originator drugs, panel (b) for bid‐winning generic drugs, panel (c) for non‐winning originator drugs, panel (d) for non‐winning generic drugs, and panel (e) for non‐equivalent generic drugs. The dots represent the observed monthly DDDc. The dashed and solid lines represent the pre‐policy and post‐policy trends, respectively, estimated by the ITS model. The vertical dashed line indicates the implementation of the NVBP policy (December 2019). CNY, Chinese Yuan; DDD, defined daily dose; DDDc, defined daily dose cost; ITS, interrupted time‐series; NVBP, National Volume‐Based Procurement.

The monthly trends in the DDDc of montelukast sodium are displayed in Figures 7, 8, 9. Table 3 presents the results of the ITS analysis, with estimates adjusted for autocorrelation using ARIMA models.

**Table 3:** Impact of the NVBP policy on the monthly DDDc of montelukast sodium in China (2019–2020).

Group	Baseline level (*β*_0_)	Baseline trend (*β*_1_)	Level change (*β*_2_)	Trend change (*β*_3_)
Nationwide	8.213^c^	0.065	−1.994^c^	−0.238
Originator drugs	9.250^c^	0.104	−4.434^c^	−0.222
Generic drugs	17.580^c^	−0.343^b^	−8.178^c^	0.230
Bid‐winning originator drugs	9.394^c^	0.096	−5.074^c^	−0.186^a^
Bid‐winning generic drugs	9.508^c^	0.070	−5.208^c^	−0.139^a^
Non‐winning originator drugs	8.885^c^	0.135^b^	1.710^c^	−0.042
Non‐winning generic drugs	16.125	0.113	3.635	−1.126
Non‐equivalent generic drugs	7.574^c^	0.035	2.214^c^	−0.091

*Note:* Estimates were derived from ITS models adjusted for autocorrelation using ARIMA specifications. Data are presented in CNY per DDD.

Abbreviations: ARIMA, autoregressive integrated moving average; CNY, Chinese Yuan; DDD, defined daily dose; ITS, interrupted time‐series; NVBP, National Volume‐Based Procurement.

^a^ *p* < 0.10,

^b^ *p* < 0.05,

^c^ *p* < 0.01.

At the national level, as shown in Figure 7, the implementation of the NVBP policy resulted in a statistically significant immediate decline in the DDDc of montelukast sodium, decreasing by 1.994 CNY/DDD (*β*_2_ = −1.994, *p* < 0.01); however, no significant change was observed in the long‐term trend (*p* > 0.1).

Regarding the comparison between originator and generic drugs (Figure 8), originator drugs experienced a statistically significant immediate decline in DDDc following the policy implementation, decreasing by 4.434 CNY/DDD (*β*_2_ = −4.434, *p* < 0.01). For generic drugs, there was already a statistically significant downward trend in DDDc prior to the policy intervention, decreasing by 0.343 CNY/DDD per month (*β*_1_ = −0.343, *p* < 0.05). Following the policy intervention, generic drugs experienced a further significant and substantial immediate decline, dropping by 8.178 CNY/DDD (*β*_2_ = −8.178, *p* < 0.01). However, neither originator nor generic drugs showed statistically significant changes in long‐term price trends after the intervention (*p* > 0.1).

At the subgroup level (Figure 9), distinct patterns were observed between bid‐winning and non‐winning drugs. For bid‐winning drugs, both bid‐winning originator and bid‐winning generic drugs experienced statistically significant immediate declines in DDDc following the policy implementation, decreasing by 5.074 CNY/DDD (*β*_2_ = −5.074, *p* < 0.01) and 5.208 CNY/DDD (*β*_2_ = −5.208, *p* < 0.01), respectively. Furthermore, both categories exhibited a long‐term downward trend that was significant at the 10% level, with bid‐winning originator drugs decreasing by 0.186 CNY/DDD per month (*β*_3_ = −0.186, *p* < 0.1) and bid‐winning generic drugs decreasing by 0.139 CNY/DDD per month (*β*_3_ = −0.139, *p* < 0.1).

Regarding non‐winning drugs, non‐winning originator drugs showed a significant pre‐policy upward trend (*β*_1_ = 0.135, *p* < 0.05) and experienced a statistically significant immediate increase in DDDc following the intervention, rising by 1.710 CNY/DDD (*β*_2_ = 1.710, *p* < 0.01), with no significant change in the long‐term trend (*p* > 0.1). No statistically significant changes were observed in the immediate level or long‐term trend for non‐winning generic drugs (*p* > 0.1). Notably, non‐equivalent generic drugs also experienced a statistically significant immediate increase in DDDc following the policy intervention, rising by 2.214 CNY/DDD (*β*_2_ = 2.214, *p* < 0.01), although no significant change was observed in the long‐term trend (*p* > 0.1).

## 4 Discussion

### 4.1 Global Significance and Overall Policy Impact

Reducing the damage to patients' health and the financial burden on families caused by asthma has become a global consensus. Currently, asthma is included in the World Health Organization Global Action Plan for the Prevention and Control of Non‐Communicable Diseases and the United Nations 2030 Agenda for Sustainable Development [3]. China's NVBP policy can provide valuable insights for reducing the economic burden of asthma treatment worldwide, contributing to the goal of “breathing freedom for all” [21].

In terms of the overall impact, the NVBP policy aims to reduce drug costs for patients by implementing a price reduction strategy through bulk purchasing [22]. This study evaluates the impact of the NVBP policy on the usage of montelukast sodium by quantifying monthly changes in the procurement volume, expenditures, and DDDc. Our results demonstrate that while the total procurement volume remained relatively stable, the policy led to a drastic reduction in total expenditures and daily costs. This suggests that the NVBP policy effectively reduced the economic burden of asthma treatment while maintaining a stable nationwide usage of montelukast sodium.

### 4.2 Procurement Volume Stability and Market Rationality

A notable finding of this study is the stability of montelukast sodium procurement volume—neither the nationwide total volume nor the volume of bid‐winning drugs showed significant changes in immediate level or long‐term trend following the policy. This stands in sharp contrast to the volume surge results generally observed in previous evaluations of the NVBP policy. Typically, the NVBP policy is expected to drive up procurement volume due to the price‐volume linkage rule. This effect has been confirmed in numerous studies covering medications for chronic diseases [14, 15, 23, 24, 25], antineoplastic agents [16, 17], and antibiotics [18], all of which observed significant increases in procurement volume.

However, the absence of such a surge in our study may be because montelukast sodium serves as a long‐term maintenance medication for asthma and allergic rhinitis; its market may have already matured prior to the NVBP, and patients are unlikely to substantially increase consumption merely due to price reductions [26]. In addition, the stable procurement volume suggests that physicians may not have engaged in irrational over‐prescription solely to meet the targets for prioritizing bid‐winning drugs. This finding provides support to counter the criticism that NVBP might induce “supplier‐induced demand” [27]. Despite concerns that mandatory targets might pressure doctors to over‐prescribe bid‐winning drugs, leading to resource waste [15], the stability observed in this study suggests that clinical usage in the respiratory chronic disease sector remains rational and driven by actual clinical need rather than policy targets.

Furthermore, comparing our results with other studies on NVBP procurement volumes during the same period helps mitigate the confounding impact of the COVID‐19 pandemic. A primary concern is whether the lack of volume increase in this study should be attributed to restricted hospital access during the pandemic. However, considering that those studies covered the identical period (January 2019 to December 2020) yet observed significant procurement volume increases for other medications [14, 16], this implies that hospital drug supply chains and patient access channels remained functional despite the pandemic. Therefore, the stability of montelukast sodium likely reflects its intrinsic usage patterns and policy effects, rather than an artifact of service disruptions caused by COVID‐19.

### 4.3 Structural Optimization: Generic Substitution and Quality Improvement

While the total procurement volume remained stable, the NVBP policy triggered a significant structural reshaping, manifested as the dual effects of “generic substitution” and “market quality optimization.” First, results show a significant immediate increase in generic drug procurement following policy implementation (*p* < 0.1), accompanied by a significant decline in non‐winning originator drugs (*p* < 0.01). This trend confirms that the NVBP policy effectively drove the “Generic Substitution Effect.” This aligns with findings from Wang et al. [28], Zhao et al. [29], and Zhao and Wu [30], indicating that by guaranteeing volume and enforcing quality standards, the NVBP successfully broke the long‐standing market dominance of originators and facilitated the clinical substitution of high‐priced originators with high‐quality generics. Second, the procurement volume of non‐equivalent generics declined significantly (*p* < 0.01). Since passing the generic consistency evaluation is the benchmark for verifying pharmaceutical and bio‐equivalence with originators, the sharp contraction of non‐equivalent generics implies a substantial reduction in low‐quality or therapeutically uncertain drugs in the market. This shift not only improves overall market quality and ensures patient safety but also creates a mechanism that compels generic manufacturers to enhance capabilities and increase research and development investment to pass the generic consistency evaluation, thereby driving high‐quality development across the pharmaceutical industry. Given this significant shift in market volume, for bid‐winning generics, the policy focus must prioritize ensuring a stable supply to meet the consolidated volume. Regulatory authorities should strengthen the monitoring of production capacity and distribution channels to prevent potential shortages driven by the rapid surge in demand.

### 4.4 Cost Containment, Bargaining Power, and Strategic Pricing

In terms of DDDc, significant immediate reductions were observed across the nationwide total, originator drugs, generic drugs, and both bid‐winning originator and generic products (*p* < 0.01). Notably, bid‐winning drugs even exhibited a significant long‐term downward trend (*p* < 0.1), which directly drove the rapid decline in the nationwide total procurement expenditure for montelukast sodium (*p* < 0.1). Real‐world market data reveal that the average price of montelukast sodium dropped from 6.39 CNY per tablet in 2018 to 5.54 CNY in 2019. The bid‐winning prices were even lower: the bid‐winning originator (MSD) dropped to 3.88 CNY, and the bid‐winning generic (Anbison) to 3.79 CNY. This provides strong evidence that the NVBP effectively lowered drug prices, significantly enhancing patient affordability and drug accessibility.

This substantial price reduction highlights the strong bargaining power inherent in the NVBP policy. These results align with findings from other global procurement practices: an analysis of pooled procurement in low‐ and middle‐income countries reported price reductions of approximately 15% [31]; Roy Chaudhury et al. noted that the “Delhi Model” pooled procurement system saved nearly 30% of the annual drug bill [32]; and Brazil successfully saved approximately $400 million and stabilized the supply chain from 2010 to 2013 through the centralized bidding mechanism established by its National Medicines Policy [33]. China's “national‐level” volume advantage played a decisive role in price negotiations, offering a valuable reference model for reducing the economic burden of asthma treatment globally.

However, the total procurement expenditure for generics increased significantly, which was likely driven by the substantial rise in procurement volume (quantity‐driven). Meanwhile, the DDDc for non‐winning originator and non‐equivalent generic drugs showed an immediate increase following the policy implementation. This may be attributed to a strategic response: as their original market share was heavily eroded by bid‐winning products due to price or quality disadvantages, manufacturers might have raised prices in the short term to maintain revenue or profit margins (Market Segmentation Strategy). However, the absence of a significant change in the long‐term trend for non‐winning drugs suggests that while short‐term strategic price rebounds occurred, this premium did not evolve into a sustained long‐term burden, indicating that these consequences remain controllable within the overall policy framework.

### 4.5 Limitations

This study has several limitations. First, the observation window is relatively short. Due to data availability, our post‐intervention period covers only 1 year (until December 2020). While the ITS analysis meets the statistical requirements for minimum data points, this limits our ability to evaluate the long‐term dynamic effects of the NVBP policy on the montelukast sodium market. Future studies with longer time series are needed to verify the sustainability of these trends. Second, this study relies on procurement data rather than patient‐level prescription or clinical outcome data. Although procurement volume is generally considered a reliable proxy for drug usage, we could not directly assess changes in patient medication adherence, asthma control levels, or the incidence of adverse events following the policy implementation. Third, the data source primarily covers public healthcare institutions. Since the NVBP policy significantly squeezed the market share of non‐winning drugs in public hospitals, these products may have shifted to retail pharmacies or private hospitals. Due to the lack of retail sector data, this study may underestimate the actual usage of non‐winning originator and non‐equivalent generic drugs in the off‐hospital market. Finally, we discuss the confounding impact of COVID‐19. Although we attempted to distinguish the policy effect from the pandemic's impact by cross‐comparing with studies in other therapeutic areas, the pandemic remains a broad external shock. Therefore, its potential residual confounding effects cannot be entirely eliminated through statistical means.

## 5 Conclusions

This study evaluated the impact of the NVBP policy on montelukast sodium using national procurement data from 24 months (January 2019 to December 2020) through ITS analysis. The results indicate that the policy successfully achieved cost containment without compromising patient access. In contrast to the “volume surge” observed in previous studies, both the total procurement volume and the volume of bid‐winning drugs remained stable. This stability suggests that procurement volume was not driven by irrational behaviors such as “supplier‐induced demand” or over‐prescription to meet policy quotas—concerns often raised in other studies—thereby reflecting the rationality and sustainability of the policy implementation.

Through a stratified analysis of originator versus generic and bid‐winning versus non‐winning drugs, we observed that the NVBP triggered significant structural optimization. The study validated a clear “generic substitution” effect, where quality‐assured, lower priced bid‐winning generics rapidly replaced high‐priced originator drugs. Furthermore, the sharp decline in the use of non‐equivalent generics indicates that the policy effectively purified the market by eliminating low‐quality products.

In addition, we further explored potential unintended consequences of the policy. Although we observed a counter‐intuitive increase in the DDDc of non‐winning drugs—suggesting that non‐winning manufacturers adopted strategic price hikes to maintain profit margins—the overall procurement expenditure nevertheless decreased substantially as expected.

Overall, the NVBP policy demonstrated its effectiveness in balancing affordability and accessibility. It successfully leveraged China's national‐level bargaining power to alleviate the economic burden of asthma treatment. Therefore, this “China Model” offers valuable insights for other countries seeking to optimize pharmaceutical spending and improve health equity in chronic disease management.

## Author Contributions

**Yufei Cheng:** conceptualization, writing – original draft, data curation, visualization, formal analysis. **Fanyu Liu:** writing – original draft, writing – review and editing, data curation, visualization, formal analysis. **Yongyi Wu:** writing – original draft, writing – review and editing, data curation, visualization, formal analysis. **Siyu Liu:** writing – original draft, writing – review and editing, data curation, visualization, formal analysis. **Xiaohan Fan:** writing – review and editing, data curation, validation. **Bin Jiang:** conceptualization, methodology, data curation, funding acquisition, writing – review and editing, resources, project administration, software, investigation. **Zhao Yang:** writing – review and editing, conceptualization, funding acquisition, methodology, data curation, resources, project administration, validation, supervision.

## Ethics Statement

The study is based on existing data from the National Healthcare Security Administration database. As the study does not involve animal or human clinical trials and does not involve personal data, it solely includes pharmaceutical procurement records from healthcare institutions. The study was approved by the Ethics Committee of Peking University (Approval No. 2025R0433‐0001).

## Consent

The Ethics Committee of Peking University approved the waiver of informed consent for this study.

## Conflicts of Interest

The authors declare no conflicts of interest.

## Data Availability

Raw data were generated at the National Healthcare Security Administration. Derived data supporting the findings of this study are available from the corresponding author on request.
